# Draft genome sequencing of a multidrug-resistant and virulent *Escherichia coli* strain isolated from milk of a cow with clinical mastitis

**DOI:** 10.1128/mra.01260-24

**Published:** 2025-02-18

**Authors:** Kh. Yeashir Arafat, Soharth Hasnat, Naim Siddique, Md. Morshedur Rahman, Syeda Fowzia Homa, M. Nazmul Hoque

**Affiliations:** 1 Molecular Biology and Bioinformatics Laboratory, Department of Gynecology, Obstetrics and Reproductive Health, Bangabandhu Sheikh Mujibur Rahman Agricultural University (BSMRAU), Gazipur, Bangladesh; Queens College Department of Biology, Queens, New York, USA

**Keywords:** draft genome, *Escherichia coli*, multidrug-resistant, virulent, clinical mastitis, dairy cow

## Abstract

We present the draft genome of *Escherichia coli* strain MBBL3, a virulent and antimicrobial-resistant pathogen isolated from the milk of a cow suffering from clinical mastitis. The assembled genome spans 4,713,190 base pairs, organized into 115 contigs, and harbors 52 antimicrobial resistance genes (ARGs) alongside 25 virulence factor genes (VFGs).

## ANNOUNCEMENT

Pathogenic strains of *E. coli* are known to cause severe infections in both humans and animals (1, 2). Among these, mammary pathogenic *E. coli* stands out as a significant causative agent of clinical mastitis (CM) in lactating mammals, particularly dairy cows (3–5). This condition causes inflammation of the udder parenchyma, leading to pathological changes in glandular tissue and disruptions in milk production, which collectively have a substantial economic impact on the global dairy industry (6, 7). In this study, we isolated and characterized a multidrug-resistant and virulent *E. coli* strain MBBL3, from the milk of a crossbred Holstein Friesian cow diagnosed with CM (8) in Gazipur district (24.09° N, 90.41° E), Bangladesh.

To isolate *E. coli* MBBL3, a 100 µL aliquot of each collected milk sample was immediately placed in 10 mL of nutrient broth upon collection and aerobically incubated overnight at 37°C (9). The enriched broth was then streaked onto Eosin-Methylene Blue (EMB) agar, and after 24 h of incubation, distinctive metallic green sheen colonies were observed. These colonies were subsequently sub-cultured on fresh EMB agar to ensure purity and eliminate contamination. The isolate was initially identified as *E. coli* based on phenotypic features *viz*. colony morphology (metallic sheen and circular shape), Gram staining (Gram −ve), and biochemical tests including indole, methyl red, citrate (IMViC), and lactose fermentation tests (4, 9, 10). For species-level identification, we used the VITEK-2 system v9.01 (VITEK 2 GN card) (11). DNA extraction and whole genome sequencing (WGS) were conducted following established protocols from previous studies (12, 13). Briefly, pure single colonies were used for DNA extraction utilizing the QIAamp DNA Mini Kit (QIAGEN, Germany). Libraries were prepared with 1 ng of DNA using the Nextera DNA Flex Kit (Illumina, USA), and sequencing was performed on an Illumina MiSeq platform employing a 2 × 250 bp paired-end read protocol (14). Genomic analysis began with quality assessment using FastQC v0.11.7 (15), followed by trimming adapters, Illumina artifacts, and phiX contamination using Trimmomatic v0.39 (16). Genome assembly was carried out using SPAdes v3.15.5 (17), and genome annotation was completed with the NCBI Prokaryotic Genome Annotation Pipeline v6.6 (18). Genome completeness was evaluated using CheckM v1.2.3 (19), ensuring a high-quality draft genome for downstream analysis. In MBBL3 genome, VFGs were predicted using the Virulence Factor Database (20), whereas ARGs were annotated with the Comprehensive Antibiotic Resistance Database version 3.2.4 (21). PlasmidFinder version 2.1 (22) was utilized to detect plasmid presence, and functional subsystem analysis was conducted via the RAST v2.0 web server (23). The potential human pathogenicity of the isolate was assessed using PathogenFinder v1.1 (24), with all tools applied under default parameters.

The key genomic characteristics of *E. coli* MBBL3 are presented in Table 1. The assembled genome spans 4.7 Mbps with a GC content of 50.5%. We predicted 52 ARGs and 25 VFGs, along with 251 functional subsystems. Additionally, the strain was linked to 105 pathogenic families and exhibited a predicted score of 0.864 (out of 1.0) for being a human pathogen. These results highlight the potential role of *E. coli* MBBL3 in CM in dairy cattle, emphasizing its relevance in veterinary and public health research.

**TABLE 1:** Genomic features of *E. coli* MBBL3 isolated from milk of a cow with clinical mastitis

Genome feature	*E. coli* MBBL3
GenBank accession	JBJCYW000000000
Assembled genome Size (bp)	4,713,190
GC %	50.5
Coverage	100.0x
Genome completeness (%)	65.75%
CheckM contamination (%)	30.07
BioSample accession	SAMN44618142
SRA accession	SRR31264072
N_50_ value (bp)	102034
Number of contigs	115
Genes (total)	4,621
CDSs (total)	4,575
Genes (coding)	4,359
CDSs (with protein)	4,359
Genes (RNA)	46
rRNAs	1 (5S)
Complete rRNAs	1 (5S)
tRNAs	34
ncRNAs	11
Pseudo genes (total)	216
CDSs (without protein)	216
Pseudo genes (ambiguous residues)	0 of 216
Pseudo genes (frameshifted)	41 of 216
Pseudo genes (incomplete)	152 of 216
Pseudo genes (internal stop)	42 of 216
Pseudo genes (multiple problems)	19 of 216
CRISPR Arrays	3
Number of ARGs	52
Number of Virulence gene	25
Number of subsystems	251
PathogenFinder score	0.864
Matched with pathogenic families	105

## Data Availability

The whole genome shotgun project of the *E. coli* strain MBBL3 has been deposited in GenBank and the NCBI Sequence Read Archive (SRA) under BioProject accession number PRJNA1182972. The versions described in this paper is version JBJCYW000000000.1.

## References

[1] Pebdeni AB, Roshani A, Mirsadoughi E, Behzadifar S, Hosseini M. 2022. Recent advances in optical biosensors for specific detection of E. coli bacteria in food and water. Food Control 135:108822. doi:10.1016/j.foodcont.2022.108822

[2] Puvača N, de Llanos Frutos R. 2021. Antimicrobial resistance in Escherichia coli strains isolated from humans and pet animals. Antibiotics (Basel) 10:69. doi:10.3390/antibiotics10010069 33450827 PMC7828219

[3] Campos FC, Castilho IG, Rossi BF, Bonsaglia ÉCR, Dantas STA, Dias RCB, Fernandes Júnior A, Hernandes RT, Camargo CH, Ribeiro MG, Pantoja JCF, Langoni H, Rall VLM. 2022. Genetic and antimicrobial resistance profiles of mammary pathogenic E. coli (MPEC) isolates from bovine clinical mastitis. Pathogens 11:1435. doi:10.3390/pathogens11121435 36558768 PMC9781227

[4] Hoque MN, Faisal GM, Jerin S, Moyna Z, Islam MA, Talukder AK, Alam MS, Das ZC, Isalm T, Hossain MA, Rahman A. 2024. Unveiling distinct genetic features in multidrug-resistant Escherichia coli isolated from mammary tissue and gut of mastitis induced mice. Heliyon 10:e26723. doi:10.1016/j.heliyon.2024.e26723 38434354 PMC10904246

[5] Hoque MN, Jerin S, Faisal GM, Das ZC, Islam T, Rahman A. 2023. Whole-genome sequence of multidrug-resistant Escherichia coli strains isolated from mice with mastitis. Microbiol Resour Announc 12:e0032023. doi:10.1128/mra.00320-23 37314348 PMC10353464

[6] Hoque MN, Rahman MS, Islam T, Sultana M, Crandall KA, Hossain MA. 2022. Induction of mastitis by cow-to-mouse fecal and milk microbiota transplantation causes microbiome dysbiosis and genomic functional perturbation in mice. Anim Microbiome 4:43. doi:10.1186/s42523-022-00193-w 35794639 PMC9258091

[7] Jha A, Hoque M, Kamal M, Rahman M, Bhuiyan M, Shamsuddin M. 2010. Prevalence of mastitis and efficacy of different treatment regimens on clinical mastitis in cows

[8] Hoque M.N, Das ZC, Talukder AK, Alam MS, Rahman ANMA. 2015. Different screening tests and milk somatic cell count for the prevalence of subclinical bovine mastitis in Bangladesh. Trop Anim Health Prod 47:79–86. doi:10.1007/s11250-014-0688-0 25326717

[9] Hoque MN, Istiaq A, Clement RA, Gibson KM, Saha O, Islam OK, Abir RA, Sultana M, Siddiki AZ, Crandall KA, Hossain MA. 2020. Insights into the resistome of bovine clinical mastitis microbiome, a key factor in disease complication. Front Microbiol 11:860. doi:10.3389/fmicb.2020.00860 32582039 PMC7283587

[10] Ievy S, Hoque MN, Islam MS, Sobur MA, Ballah FM, Rahman MS, Rahman MB, Hassan J, Khan MFR, Rahman MT. 2022. Genomic characteristics, virulence, and antimicrobial resistance in avian pathogenic Escherichia coli MTR_BAU02 strain isolated from layer farm in Bangladesh. J Glob Antimicrob Resist 30:155–162. doi:10.1016/j.jgar.2022.06.001 35671989

[11] Kumaran D, Laflamme C, Ramirez-Arcos S. 2023. A multiphasic approach to solve misidentification of Cutibacterium acnes as Atopobium vaginae during routine bacterial screening of platelet concentrates using the VITEK 2 system. Access Microbiol 5:000539. doi:10.1099/acmi.0.000539.v3 PMC10323807 37424557

[12] Rahman MM, Siddique N, Akter S, Das ZC, Hoque MN. 2024. Draft genome sequencing of Pediococcus pentosaceus strains isolated from cow milk. Microbiol Resour Announc 13:e0023824. doi:10.1128/mra.00238-24 38619270 PMC11080533

[13] Rahman MM, Siddique N, Rahman AA, Das ZC, Islam T, Hoque MN. 2024. Whole-genome sequencing of Enterococcus faecalis probiotic strains isolated from raw milk of healthy cows. Microbiol Resour Announc 13:e0046524. doi:10.1128/mra.00465-24 39162453 PMC11385455

[14] Sultana T, Siddique N, Rahaman M, Rahman MM, Rahman AA, Talukder AK, Das ZC, Hoque MN. 2024. Draft genome sequencing of multidrug-resistant Pseudomonas asiatica strains isolated from dairy cows with clinical mastitis and their farm environment. Microbiol Resour Announc 13:e0090724. doi:10.1128/mra.00907-24 39404440 PMC11556121

[15] Andrews S. 2010. FastQC: a quality control tool for high throughput sequence data. Babraham Bioinformatics, Babraham Institute, Cambridge, United Kingdom.

[16] Bolger AM, Lohse M, Usadel B. 2014. Trimmomatic: a flexible trimmer for Illumina sequence data. Bioinformatics 30:2114–2120. doi:10.1093/bioinformatics/btu170 24695404 PMC4103590

[17] Bankevich A, Nurk S, Antipov D, Gurevich AA, Dvorkin M, Kulikov AS, Lesin VM, Nikolenko SI, Pham S, Prjibelski AD, Pyshkin AV, Sirotkin AV, Vyahhi N, Tesler G, Alekseyev MA, Pevzner PA. 2012. SPAdes: a new genome assembly algorithm and its applications to single-cell sequencing. J Comput Biol 19:455–477. doi:10.1089/cmb.2012.0021 22506599 PMC3342519

[18] Tatusova T, DiCuccio M, Badretdin A, Chetvernin V, Nawrocki EP, Zaslavsky L, Lomsadze A, Pruitt KD, Borodovsky M, Ostell J. 2016. NCBI prokaryotic genome annotation pipeline. Nucleic Acids Res 44:6614–6624. doi:10.1093/nar/gkw569 27342282 PMC5001611

[19] Parks DH, Imelfort M, Skennerton CT, Hugenholtz P, Tyson GW. 2015. CheckM: assessing the quality of microbial genomes recovered from isolates, single cells, and metagenomes. Genome Res 25:1043–1055. doi:10.1101/gr.186072.114 25977477 PMC4484387

[20] Chen L, Zheng D, Liu B, Yang J, Jin Q. 2016. VFDB 2016: hierarchical and refined dataset for big data analysis—10 years on. Nucleic Acids Res 44:D694–D697. doi:10.1093/nar/gkv1239 26578559 PMC4702877

[21] Jia B, Raphenya AR, Alcock B, Waglechner N, Guo P, Tsang KK, Lago BA, Dave BM, Pereira S, Sharma AN, Doshi S, Courtot M, Lo R, Williams LE, Frye JG, Elsayegh T, Sardar D, Westman EL, Pawlowski AC, Johnson TA, Brinkman FSL, Wright GD, McArthur AG. 2017. CARD 2017: expansion and model-centric curation of the comprehensive antibiotic resistance database. Nucleic Acids Res 45:D566–D573. doi:10.1093/nar/gkw1004 27789705 PMC5210516

[22] Carattoli A, Zankari E, García-Fernández A, Voldby Larsen M, Lund O, Villa L, Møller Aarestrup F, Hasman H. 2014. In silico detection and typing of plasmids using PlasmidFinder and plasmid multilocus sequence typing. Antimicrob Agents Chemother 58:3895–3903. doi:10.1128/AAC.02412-14 24777092 PMC4068535

[23] Overbeek R, Olson R, Pusch GD, Olsen GJ, Davis JJ, Disz T, Edwards RA, Gerdes S, Parrello B, Shukla M, Vonstein V, Wattam AR, Xia F, Stevens R. 2014. The SEED and the rapid annotation of microbial genomes using subsystems technology (RAST). Nucleic Acids Res 42:D206–D214. doi:10.1093/nar/gkt1226 24293654 PMC3965101

[24] Cosentino S, Voldby Larsen M, Møller Aarestrup F, Lund O. 2013. PathogenFinder--distinguishing friend from foe using bacterial whole genome sequence data. PLoS One 8:e77302. doi:10.1371/journal.pone.0077302 24204795 PMC3810466

